# Editorial: Early life events: shedding light on neurobiological mechanisms

**DOI:** 10.3389/fnbeh.2023.1209494

**Published:** 2023-05-17

**Authors:** Randriely Merscher Sobreira de Lima, Natividade de Sá Couto Pereira, Carla Dalmaz, Danusa Mar Arcego

**Affiliations:** ^1^Department of Psychiatry, McGill University, Montreal, QC, Canada; ^2^Douglas Mental Health University Institute, McGill University, Montreal, QC, Canada; ^3^Psychological Neuroscience Laboratory, Psychology Research Centre, School of Psychology, University of Minho, Braga, Portugal; ^4^Departamento de Bioquímica, Instituto de Ciências Básicas da Saúde, Universidade Federal do Rio Grande do Sul, Porto Alegre, Brazil

**Keywords:** early life adversity, stress, development, child development, early environment

Early life experiences may impact the development, affecting neural, behavioral, and psychological domains (Kessler et al., 2010; Couto-Pereira et al., 2019). These experiences can shape individual differences in neural plasticity and behavior with lifelong effects. Environmental conditions present in the early perinatal and prepubertal periods have a substantial influence on an individual's susceptibility or resilience, as these periods undergo critical changes during this time (Belsky and Pluess, 2013). The effects of adverse conditions in early life have been observed to affect the development of brain functioning and other biological systems, increasing vulnerability to diseases and behavioral changes later in life (de Lima et al., 2020). Although many studies recognize the relationship between the quality of the early environment and the influence on multiple aspects of child development, little is known about the neurobiological mechanisms underlying this association. A deeper understanding of this interplay will shed light on the neurobiological basis of an individual's long-term vulnerability to disease. By gathering innovative contributions in this area, we aimed to advance the knowledge of how early environments lead to different phenotypes concerning vulnerability or resilience to stress later in life. The contributions comprising this special issue cover a range from animal models to translational papers studying children's behavior and brain's functional connectivity, as depicted in Figure 1.

**Figure 1:**
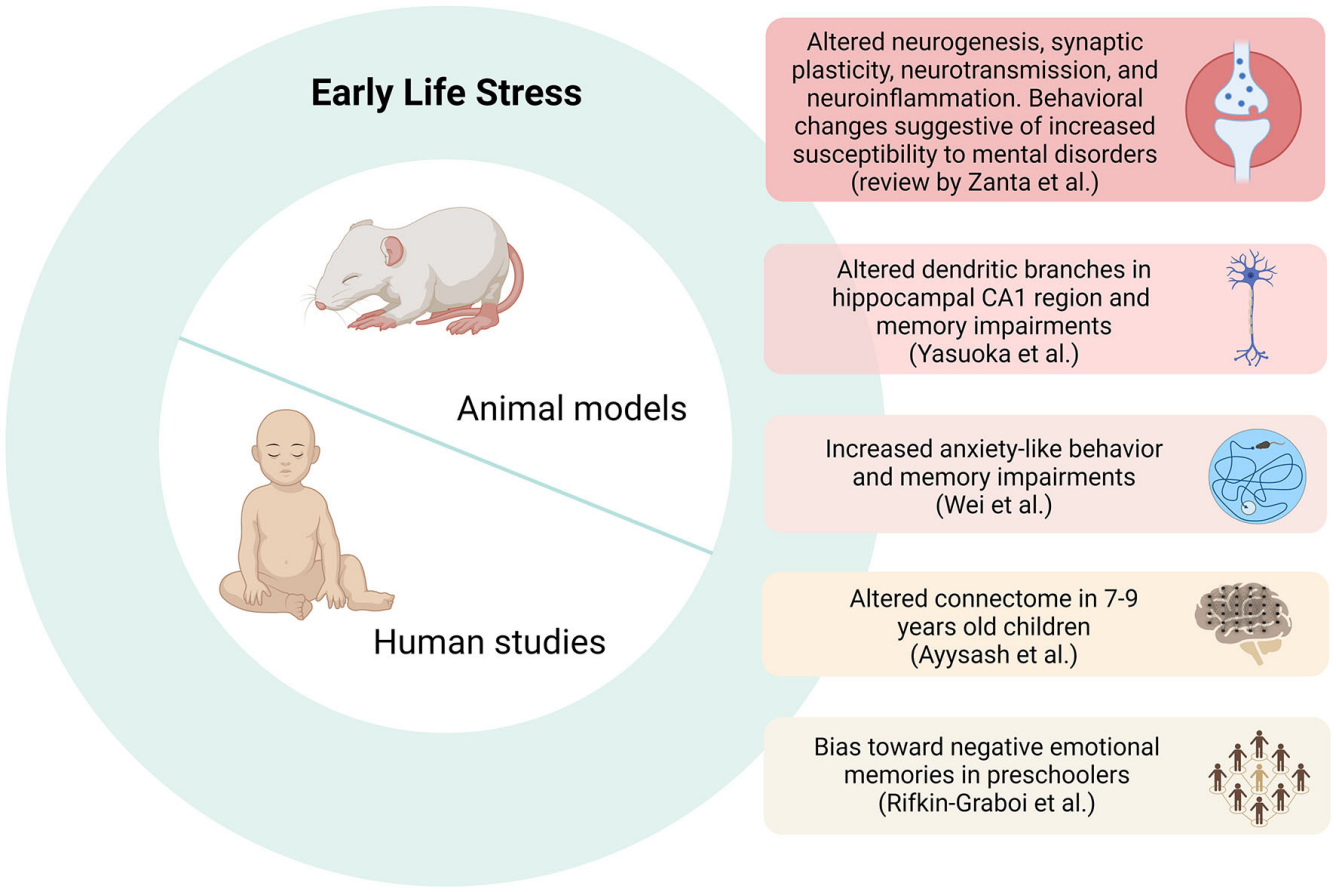
The influence of early life environment on brain development. The figure summarizes the studies that composed the Research Topic: *Early Life Events: Shedding Light on Neurobiological Mechanisms*.

In *Neurobiological mechanisms involved in maternal deprivation-induced behaviors relevant to psychiatric disorders*, Zanta et al. provides an updated appraisal of the effects of maternal deprivation, an animal model of parental loss. This procedure may lead to increased susceptibility in animal models of mental disorders, in particular related to depression, anxiety, and schizophrenia, and the authors argue for some putative mechanisms that could explain the behavioral outcomes. That sex-dimorphic effects were observed in most of the neurobiological outcomes is of indisputable importance. The review covers from immediate effects to long-term outcomes involving the hypothalamic-pituitary-adrenal axis, neurogenesis and neuroplasticity, neurotransmitter, neuromodulatory systems, and neuroinflammation, relating effects on these processes with the observed behavioral changes.

Two studies consider different animal models of variant early environments and their influence on cognition. *Mastication stimuli enhance the learning ability of weaning-stage rats, altering the hippocampal neuron transcriptome and micromorphology*, conducted by Yasuoka et al. sheds light on the correlation between masticatory stimulation and memory using powdered or solid diets during a sensitive period of development. The study investigates neural interaction between mastication stimuli and memory processing, hippocampal transcriptome, and dendrite morphology. The results reveal that mastication stimuli during the weaning period have a positive effect on rat's memory function, probably through the increase of dendrite branches of hippocampal CA1 neurons and gene regulation related to dendrite growth. These findings underscore the importance of adequate mastication during developmental periods for proper brain function.

The second study, *Altered cognition and anxiety in adolescent offspring whose mothers underwent different-pattern maternal sleep deprivation, and cognition link to hippocampal expressions of Bdnf and Syt-1*, is based on the evidence that sleep disturbance during pregnancy may negatively affect the development of offspring. Wei et al. investigated the association between sleep deprivation in late pregnancy with emotion, cognition, and expression of synaptic plasticity-related proteins in offspring mice. Pregnant mice were used for studying the different patterns and durations of sleep deprivation. The results demonstrated that maternal sleep deprivation during late pregnancy impairs emotion and cognition in offspring, which is associated with altered hippocampal *bdnf* and *syt1* expression.

Two studies examined exposure to early adversities in children. In *Examining resting-state network connectivity in children exposed to perinatal maternal adversity using anatomically weighted functional connectivity (awFC) analyses; A preliminary report*, Ayyash et al. explored adversity-related changes using an innovative approach to brain imaging analysis, that combines functional and structural connectivity, the awFC. Compared to controls, children whose mothers had poor mental health and/or low socioeconomic status during the perinatal period exhibited lower connectivity in some cortical and limbic networks, but increased connectivity in the ventral attention network. The awFC approach highlighted the importance of accounting for structural aspects when assessing functional connectivity in developmental networks. This study provides further support that early life stress impacts the course of brain maturation, specifically the developmental tuning of functional and structural connectivity.

Early life experiences are the basis of a child's health during development and early life caregiving adversity may influence future negative memory biases. The study *Variation in maternal sensitivity and the development of memory biases in preschoolers* by Rifkin-Graboi et al. explored whether insensitive care predicts subsequent memory biases for threatening stimuli in preschoolers, and if such relations could influence different forms of memories and hippocampal volume. Using data from the Two Wave Study, the authors showed that exposure to insensitive caregiving predicted the difference between emotional memories which were associated with hippocampal body volumes. The study highlights that lower levels of maternal sensitivity when children were four and a half years of age predicted better relational memory when angry stimuli were involved.

These studies underline the need for additional research focusing on the effects of stress during early developmental stages, when brain circuits are still maturing and adapting, in order to better understand how stress imprints into neural circuits and changes their functioning throughout life. In the future, this understanding of the mechanisms associated with early life adversity may help to better establish protective strategies. In addition, filling the knowledge gap on sexual dimorphism in the neurobiological mechanisms of early life stress, as well as how protective strategies may reverse several of those effects will allow the development of more accurate treatments for a set of psychiatric disorders.

## Author contributions

RMSL, NSCP, CD, and DMA wrote, reviewed, and edited the editorial manuscript. All authors contributed to the article and approved the submitted version.
